# How to Speak

**Published:** 1891-10

**Authors:** 


### HOW TO SPEAK.

The work of teaching children how to speak correctly belongs primarily to the home life, and is as surely one of its duties as any thing else pertaining to the household. Of course, the first obligation rests upon the parents themselves to be patterns of correct speech before they try to develop it in the children. Be sure they hear the English language spoken clearly, pleasantly, and accurately at the table and the fireside, and unconsciously they will become accustomed to expressing their thoughts in much the same style. It is well to remember that as soon as a child begins to frame its mouth for talking, direct training in the art should be aimed at. There seems to be a notion in some families that a little child cannot be petted and spoken to in a common-sense fashion, and, as a rule, the baby of the home is addressed by those around it in terms which can only be described as idiotic. Because the tiny tongue cannot successfully grapple with the letters *b* and *r*, those who should be guiding and encouraging its first efforts descend to its level, and you may find them chattering away as childishly as the baby. Thus he is deprived of a fair chance of hearing the words and letters rightly pronounced, and his progress towards perfection is so much the slower. During the first four or five years of his life a child should be addressed in pure Anglo-Saxon speech—words of one or two syllables—given with the right inflection and emphasis, so that a solid foundation of simple but intelligent utterance may be laid. After that words of foreign derivation may be introduced, but the more these are dabbled with the greater is the need of attention and caution. But correct speaking not only implies right pronunciation of words, but also their proper disposal, and here the hint may also serve for the "children of a larger growth," who oftimes display a wanton carelessness in clothing their ideas and giving expression to them. Much of the beauty and power of our language is lost because we persist in the habit of using the first term that comes to our lips, whether it be appropriate or not. The young people should also be encouraged to talk in a pleasant tone, avoiding harshness and abruptness, and at the same time the drawling and mumbling which characterize the speech of some children. I am aware that work of this kind requires teaching ability of no mean order on the part of those who attempt it, yet, patience and steady application will accomplish wonders upon the minds and habits of the young. Those parents living in the country, in little villages and hamlets where an almost unintelligible dialect prevails, will suffer the greatest discouragement. Every time the children leave the doorstep they will hear rough, uncouth phrases which have all the charm of newness and oddity to them, and they will return home and startle you with the ease and gusto with which they repeat them. It is an evil which cannot be absolutely prevented so long as you live in the neighborhood where the custom obtains, but in the every day home conversation, both by old and young, there ought to be studied and sustained effort to speak the English language in an accurate and pleasing manner.—G. Y.

